# Pre-clinical left ventricular myocardial remodeling in patients with Friedreich’s ataxia: A cardiac MRI study

**DOI:** 10.1371/journal.pone.0246633

**Published:** 2021-03-26

**Authors:** Karen A. G. Takazaki, Thiago Quinaglia, Thiago D. Venancio, Alberto R. M. Martinez, Ravi V. Shah, Tomas G. Neilan, Michael Jerosch-Herold, Otávio R. Coelho-Filho, Marcondes C. França

**Affiliations:** 1 Division of Medicine, Section of Neurology, Department of Neurology, School of Medical Sciences, University of Campinas – UNICAMP, Campinas, São Paulo, Brazil; 2 Division of Medicine, Section of Cardiology, Department of Internal Medicine, Division of Medicine, Section of Neurology, School of Medical Sciences, University of Campinas – UNICAMP, Campinas, São Paulo, Brazil; 3 Division of Medicine, Section of Cardiovascular Imaging Research Program and Cardiac MR PET CT Program, Department of Cardiology, Massachusetts General Hospital, Harvard Medical School, Boston, Massachusetts, United States of America; 4 Division of Medicine, Section of MRI Physics, Department of Radiology, Brigham and Women’s Hospital and Harvard Medical School, Boston, Massachusetts, United States of America; Univeristy of Tennessee, UNITED STATES

## Abstract

**Background:**

Heart Failure (HF) is the most common cause of death in Friedreich’s ataxia (FRDA), an inherited mitochondrial disease. Myocardial fibrosis and myocardial hypertrophy are well-documented autopsy features among FRDA patients with HF.

**Objectives:**

To leverage the unique tissue characterization features of cardiac magnetic resonance (CMR) for characterizing myocardial remodeling in patients with genetically confirmed FRDA without HF and preserved left ventricular ejection fraction (LVEF > 55%).

**Methods:**

Twenty-seven FRDA’s patients (age 27.6 ± 9.7 years, 15 women) and 10 healthy controls (32.6±7.3 years, 5 women) underwent a CMR for assessment of LV function, myocardial T1, late gadolinium enhancement (LGE), extracellular volume fraction (ECV), and intracellular water-lifetime (τ_ic_), a marker of cardiomyocyte size.

**Results:**

As compared to controls, FRDA patients had a preserved LVEF (LVEF: 70.5±7.4% vs. 63.9±9.0%, P<0.058), larger LV mass index (LVMASSi: 61±21.7 vs. 45±4.2g/m^2^, P<0.02), and decreased LV end-diastolic volume index (LVEDVi 53.1±12.0 vs. 75.7±16.1ml/m^2^, P<0.001), compared with controls. Additionally, ECV and cardiomyocyte size (τ_ic_,) were larger in FRDA patients (ECV: 0.36 ±0.05 vs. 0.25±0.02, P<0.001; τ_ic_: 0.15±0.08 vs. 0.06±0.03 s, P = 0.02). ECV and τ_ic_ were positively associated with LV mass-to-volume ratio (ECV: r = 0.57, P = 0.003; τ_ic_: r = 0.39; P = 0.05). LVMASSi and cardiomyocyte mass-index [(1−ECV)·LVMASSi] declined with age at the CMR exam, independent of the age at initial diagnosis.

**Conclusions:**

LV hypertrophy and concentric LV remodeling in FRDA are associated at the tissue level with an expansion of the ECV and an increase in cardiomyocyte size. The adverse tissue remodeling assessed by ECV and τ_ic_ is associated with more severe cardiomyopathy classification, suggesting a role for these markers in tracking disease progression.

## Introduction

Friedreich’s ataxia (FRDA) is the most frequent autosomal recessive ataxia worldwide with a prevalence of approximately 1 in 50,000 [1]. It is caused by homozygous GAA expansions in intron 1 of the *FXN* gene on chromosome 9q13 [2]. The mutation results in a severe deficiency of frataxin, a mitochondrial protein that plays a key role in iron homeostasis [3]. This leads to nervous system, endocrine and cardiac damage. Neurodegeneration in FRDA extends to dorsal root ganglia, spinal cord, and deep cerebellar nuclei [4]. Thus, mixed sensory and cerebellar ataxia combined with pyramidal signs are the major neurological characteristics of FRDA.

Cardiac involvement is found in up to 60% of all FRDA patients and it is considered the leading cause of death [5]. It consists of concentric and symmetrical increase of ventricular wall thickness associated with either a normal or small left ventricular (LV) cavity [6,7]. While systolic function is usually preserved early in the disease course, cardiac systolic dysfunction ultimately takes place in late stages, leading to congestive heart failure (HF) and ventricular arrhythmias [8]. Proliferation of myocardial interstitial fibrosis is a histological hallmark of established FRDA’s cardiomyopathy with clinical HF [9], and constitute the tissue substrate for arrhythmias and HF.

Cardiac magnetic resonance (CMR) imaging has emerged as a promising diagnostic technique in FRDA patients, not only to determine the severity of LV remodeling [10,11], but also to assess the expansion of interstitial myocardial fibrosis (e.g., myocardial T1 time and extracellular volume fraction, ECV) and cardiomyocyte size [12–14]. These novel biomarkers provided valuable insights into the myocardial microstructural abnormalities in several diseases, and their potential for following heart disease in the long term has been highlighted in recent recommendations [12]. Nevertheless, they have not been fully explored in patients with FRDA. Therefore, in this study we investigated the myocardial extracellular volume fraction (ECV) and intracellular water-lifetime (τ_ic_), using T1-weighted CMR imaging, in a cohort of patients with FRDA without signs of HF. We also investigated whether myocardial tissue phenotyping by CMR can highlight particular characteristics of LV remodeling in FRDA’s cardiomyopathy, beyond those currently assessed with Weidemann’s imaging-based classification [15] of disease severity.

## Methods

### Subjects’ selection and clinical assessment

We recruited 27 consecutive patients with genetic confirmation of FRDA regularly followed at the University Hospital of the State University of Campinas (UNICAMP), Brazil between 2014 and 2018. All enrolled patients were homozygous for GAA expansions at *FXN*. As a control population, 10 volunteers were prospectively recruited under an IRB-approved protocol and reported elsewhere [16,17]. The current study was in accordance with the Helsinki Declaration. The Institutional Review Board at UNICAMP approved the protocol for FRDA patients (CAA: 20706412.2.0000.5404), and all participants provided informed consent. For each FRDA patient, we collected data on age at disease onset and presence of either neurological or cardiovascular symptoms. Patients underwent routine clinical and neurological examination. The standard Friedreich’s Ataxia Rating Scale (FARS.3) [18] was employed to quantify neurological function, with a higher FARS.3 score indicating worse functional status. Anthropometric data were also obtained (height, weight and BMI). On the day of CMR imaging, we also collected blood samples to determine glomerular filtration rate and hematocrit. We employed the recommendations published by Weidemann [15] to define 4 grades of the cardiomyopathic phenotype related to FRDA (FRDA-CM), which take into account two imaging parameters: end-diastolic wall thickness of the interventricular septum (IVS) and LVEF. The FRDA cardiac phenotype categories were labeled as “absent”, “mild”, “intermediate”, and “severe”.

### Cardiac magnetic resonance imaging

All subjects underwent CMR imaging in supine position in a 3-Tesla magnet (Achieva, Philips Medical Systems, Best, The Netherlands). Electrocardiographically gated cine imaging with steady state free-precession (SSFP) using repetition time TR = 3.4 ms, echo time TE = 1.5 ms, and in-plane spatial resolution = 1.5–2 mm was performed to assess left ventricular (LV) volumes, function and mass. An inversion-recovery-prepared, gradient-echo sequence with segmented acquisition was applied for late gadolinium enhancement (LGE) imaging at 10 minutes after the end of a cumulative administration of 0.2 mmol/kg of gadoterate meglumine (Dotarem, Guerbet, Aulnay-sous-Bois, France). Slice locations for LGE imaging (8-mm slice thickness) matched those for cine imaging. For the T1 quantification we used Look-Locker imaging [15] with a non-slice-selective adiabatic inversion pulse, followed by segmented gradient-echo acquisition for 17–25 times-after-inversion (TE = 2.7 ms; TR = 5.5 ms; flip angle = 10^0^; 192 x 128 matrix; 8-mm slice), covering approximately 2 cardiac cycles (TI increments: 100 ms pre-contrast, and 55 ms post-contrast, Look-Locker cycle repetition intervals>4 RR intervals pre-contrast, and 3 RR intervals post-contrast) in two short-axis slices at the level of the mid segments of the LV. In the same locations, T1 imaging was done before, and 4 to 5 times after the injection of gadolinium to cover an approximately 30-min period of contrast clearance. Volunteers were also scanned using a 3-Tesla system (Siemens Trio, Siemens Medical Systems, Erlangen, Germany) using the same techniques as for FRDA patients.

### Quantification of LV function, fibrosis and cardiomyocyte hypertrophy

Standard methods were applied to measure LV mass, volumes and function (Mass Research, Leiden University Medical Center, Leiden, the Netherlands). For each Look-Locker image series, the endocardial and epicardial borders of the LV were traced, excluding papillary muscles [19], and the LV wall was divided into 6 segments. To determine T1*, signal intensity versus time-after-inversion curves for each myocardial segment, and the blood pool, were iteratively phase-restored, and fit with a nonlinear, least-squares algorithm to the standard equation for inversion recovery. T1 was calculated from T1* and the amplitude parameters from the fit, to correct for the effects of radiofrequency pulses applied during the Look-Locker read-outs. R1 (1/T1) values for myocardial tissue and blood data were fit with a 2-space model of transcytolemmal water exchange as previously described [20]. Myocardial segments with positive LGE were excluded from the T1 mapping analysis. ECV and τ_ic_, a cell size-dependent parameter, were adjustable parameters of this model. Blood hematocrit was a fixed parameter of the model.

### Statistical analysis

Results are summarized as mean±standard deviation. T-tests were used to compare continuous variables between groups. Binary data was compared using Fisher exact test when appropriate, or a *χ2* test otherwise. Bivariate correlation was used to assess associations (Spearman or Pearson, when appropriate). Multi-variate linear regression analysis was used to build a model for τ_ic_, with LV mass index and age at disease onset as simultaneous predictors. A variance inflation factor < 3 was used as criterion as guard against multicollinearity. The Cochran Armitage test was used to test for a trend in the proportion of patients with ECV > median across the levels of the Weidemann classification of FRDA cardiac phenotype. Based on the previously described variability of ECV in healthy volunteers [16], the present sample size of 27 FRDA patients and 10 healthy volunteers provided 85% power to detect a 5% difference in mean ECV between these groups at α = 0.05 level. Statistical analyses were performed with R (version 4.0.2, The R Foundation for Statistical Computing, Vienna, Austria; http://www.Rproject.org). A two-sided p-value less than 0.05 was considered statistically significant.

## Results

### Baseline characteristics of the study population

Demographic, clinical and laboratory data of all recruited patients and controls are illustrated in Table 1. Mean age of FRDA’s patients and disease duration were 27.6±9.7 and 12.8±7.9 years, respectively. Twenty-four patients had classical FRDA presentation, while 3 had late onset FRDA (onset when older than 25 years). Mean lengths of GAA1 (longer) and GAA2 (shorter) alleles were 1051±306 and 910±254 repeats, respectively. Six (22%) patients had diabetes mellitus, one (4%) had hypertension, and none had been previously diagnosed HF. Additionally at the time of CMR, all FRDA patients were free of signs of HF with no restriction to ordinary physical activity [21].

**Table 1:** Demographic and clinical data of FDRA patients and healthy controls. All results are shown either as mean ± SD or percentages.

Demographics, clinical and laboratory data at baseline			
	FRDA (n = 27)	Healthy Controls (n = 10)	P-value
Age (years)	27.6 ± 9.7	32.6±7.3	0.145
Gender (M/F)	12/15	5/5	0.89
Systolic BP (mmHg)	116±13	114±12.9	0.62
Diastolic BP (mmHg)	74±10	72±5.3	0.44
Heart rate (bpm)	87±11.5	63±9.9	<0.001
BMI (kg/m^2^)	21.4± 3.4	24.4±4.4	0.036
Body surface (m^2^)	1.66±0.23	1.85±0.19	0.04
**Comorbidities**			
Hypertension, %, (N)	4%, (1)	0	0.89
Diabetes, %, (N)	22%, (6)	0	0.16
Hb A1C %	6.66±1.66	-	-
Fasting glucose (mg/dL)	99.3±38.4	-	-
Hyperlipidemia, % (N)	0	0	NS
Tobacco current use, %, (N)	0	0	NS
Heart Failure, %, (N)	0	0	NS
**Medication use**			
Angiotensin-converting enzyme inhibitor or angiotensin converting enzyme inhibitors, %, (N)	18%, (5)	0	0.56
Aspirin, %, (N)	0	0	NS
β-Blocker, %, (N)	4%, (1)	0	0.9
Statin, %, (N)	0	0	NS
Metformin, %, (N)	11%, (3)	0	0.56
Insulin, %, (N)	11%, (3)	0	0.56
Diuretics, %, (N)	0	0	NS
**Laboratory analyses**			
GFR	121.2±41	124.5±19.9	0.627
Hct	41.6±2.8	42.3±2.6	0.305

Data are presented as mean ± SD. MBP: Mean blood pressure; BMI: Body mass index; GFR: Glomerular filtration rate; Hct: Hematocrit, NS = non-significant, NA = not available.

Regarding neurological impairment, median FARS.3 score was 60 (range: 23–97). In this cohort, neurological function, expressed by FARS.3 scores, was strongly associated with time since disease onset (r = 0.67, P<0.001). Male patients had a significantly worse neurological status than females (74.2±16.2 vs. 48.5±16.1; p<0.001). Patients with diabetes mellitus had a worse (i.e. higher) FARS.3 score than non-diabetic patients (85.2±9.41 vs. 56.8±18.2; p<0.001). The difference in FARS.3 between diabetic and non-diabetic subjects remained significant with simultaneous adjustment by age.

### CMR Results: FRDA compared to healthy controls

**Fig 1:**
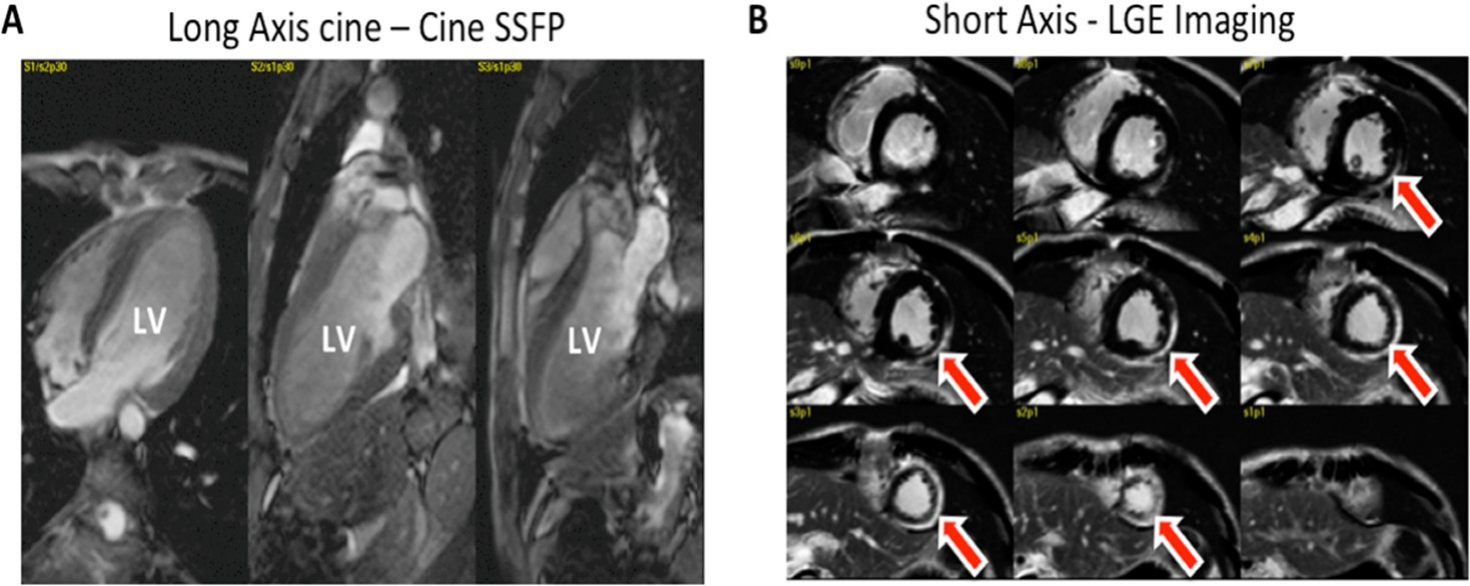
A-B: CMR images from a 37 year-old FRDA patient with LV hypertrophy and preserved LVEF (A: Diastolic cine SSFP images in long axis views) and LGE with epicardial pattern in the lateral wall of the LV (B, arrows). LV: left ventricle and Ao: aorta.

All FRDA patients completed the CMR studies without any adverse reactions. The images in Fig 1 illustrate the typical cardiac phenotype in FRDA with increased LV mass, preserved LVEF, and epicardial LGE. The main CMR findings for the FRDA and control groups are summarized in Table 2. While the FRDA group demonstrated preserved LVEF (70.5±7.4%), LV volumes and IVS thickness were all significantly increased compared to controls. Both myocardial ECV (0.36±0.05 vs. 0.25±0.02, P<0.001) and τ_ic_ (0.15 ± 0.08 s vs. 0.06±0.03 s, P = 0.02) were significantly higher in the FRDA group compared to healthy controls. In the FRDA group, τ_ic_ correlated positively with age (ρ = 0.51, P = 0.015), and τ_ic_ was significantly lower in patients with diabetes mellitus (DM) compared to non-diabetic subjects (0.096±0.058 vs. 0.172±0.08 s, P = 0.03). This difference of τ_ic_ with respect to DM remained significant (P = 0.004) with simultaneous adjustment of τ_ic_ by age (coefficient in multi-variate linear regression model for age: 0.0057 ± 0.0014; P = 0.001). Four out of 27 patients (15%) had positive LGE with an atypical pattern for coronary artery disease, comprising the mid inferolateral (n = 2) and the apical lateral segment (n = 2), whereas none of the controls presented such finding. Mid inferolateral segments in two patients showing LGE and matching with segments in T1 imaging were excluded from T1 mapping analysis.

**Table 2:** Cardiac magnetic resonance (CMR) findings in patients with Friedreich’s ataxia and healthy controls.

	FRDA (n = 27)	Healthy Controls (n = 10)	P-value
IVS thickness (mm)	13.0±3.4	7.0±0.7	<0.001
LVEF (%)	70.5±7.4	63.9±9.0	0.058
LVEDV index (ml/m^2^)	53.1± 12.0	75.7±16.1	0.001
LVESV index (ml/m^2^)	15.9±10.0	27.7±10.2	0.005
LV mass index (g/m^2^)	61.1±21.7	45.0 ± 4.2	0.002
LV mass / volume (g/ml)	1.2±0.3	0.54±0.1	<0.001
ECV	0.36 ± 0.05	0.25±0.02	<0.001
Intracellular lifetime of water (τ_ic_)	0.15±0.08	0.06±0.03	<0.001
Presence of LGE, % (N)	15% (4)	0	0.57
LGE as % of LV mass	2.25±1.7	0	<0.001
LGE (g)	5.7±4.8	0	<0.001

Data are presented as mean ± SD. P-values are for T-test statistic, or test of proportions in the case of categorical variables. IVS—Intraventricular septum, LVEF- Left ventricle ejection fraction, LVEDV- Left ventricle end diastolic volume, LVESV- Left ventricle end systolic volume, LGE—Late gadolinium enhancement.

### Associations of CMR and clinical characteristics of FDRA

**Fig 2:**
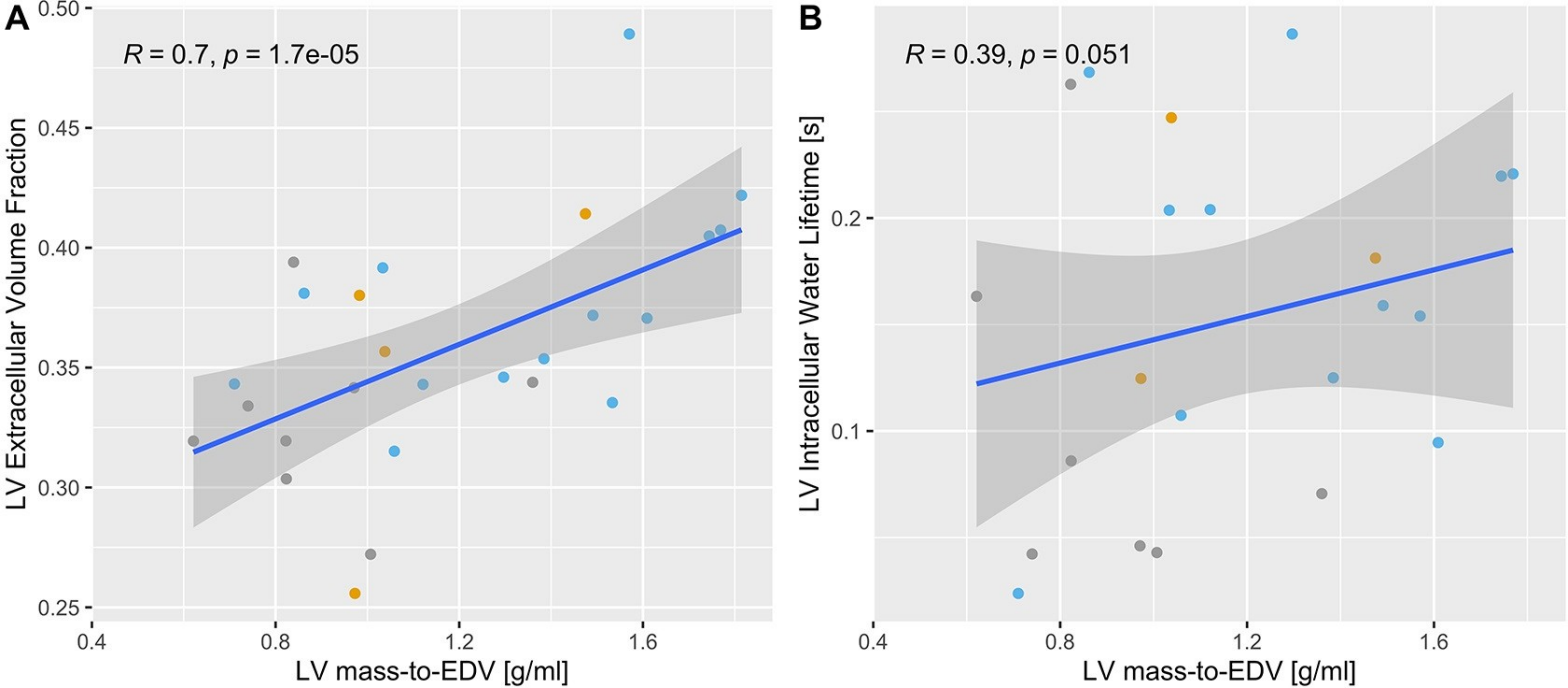
A-B: The LV mass-to-EDV (end-diastolic-volume) ratio in FRDA patients was significantly higher than in controls, indicative of a hypertrophic phenotype. In FRDA, both the extracellular volume fraction and the intracellular lifetime of water were positively associated with the LV mass-to-EDV ratio, suggesting that a build-up of interstitial fibrosis and cardiomyocyte hypertrophy contribute to the observed hypertrophic phenotype. The color of data points identifies the Weidemann’s cardiomyopathy classification (gray = absent, orange = mild and blue = intermediate).

CMR-derived global parameters of myocardial remodeling, such as LV end systolic and diastolic volumes, mass, and mass-to-volume ratios, were noticeably different between FRDA patients and controls (Table 2). LV mass index trended lower with age at CMR exam (r = -0.34, p = 0.09). ECV was positively associated with the LV mass-to-EDV (g/ml) (r = 0.7, p<0.001, Fig 2A), and intracellular water lifetime trended higher with LV mass-to-EDV (r = 0.39; P = 0.051, Fig 2B).

### CMR phenotype of FRDA and Weidemann classification

**Fig 3:**
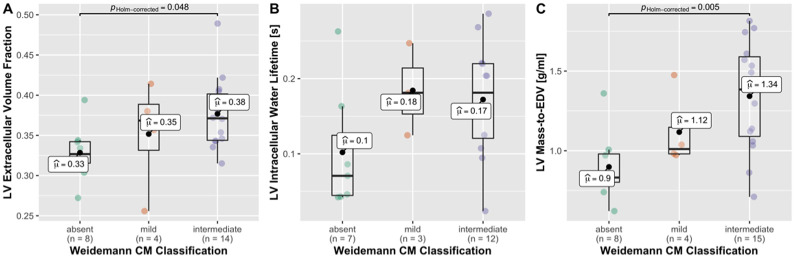
a) ECV increased significantly across Weidemann categories (P = 0.027 for linear trend; P = 0.053 for Kruskal-Wallis test) and ECV was significantly higher with the severe form of CM, compared to the group with absent signs. b) the intracellular water lifetime trended higher across Weidemann categories (P = 0.07 for linear trend); c) the degree of LV hypertrophic remodeling, assessed by the LV mass-to-EDV (end-diastolic-volume) ratio, increased across Weidemann categories, similar to the tissue markers ECV and intracellular lifetime in (a) and (b), suggesting that the changes at the tissue level are linked to the hypertrophic phenotype, based also on the results shown in Fig 2. The color of data points identifies the Weidemann’s cardiomyopathy classification (green = absent, red = mild and purple = intermediate).

According to Weidemann’s criteria for the FRDA-CM cardiac phenotype [15], 8 (30%) patients showed no signs of FRDA-CM, 4 (15%) were classified as mild FRDA-CM, 15 (55%) as intermediate FRDA-CM, and none as severe FRDA-CM. ECV increased significantly across Weidemann categories (P = 0.027 for linear trend; P = 0.053 for Kruskal-Wallis test), and ECV was significantly higher with the severe form of CM, compared to the group with absent signs (Fig 3A). The proportion of FRDA patients with “mild” or “intermediate” signs of FRDA CM increased significantly across tertiles of ECV (P = 0.031). Signs of FRDA-CM were predominantly absent in the lowest ECV tertile (P<0.05 for test of proportions in lowest ECV tertile). The intracellular water lifetime trended higher across Weidemann categories (P = 0.07 for linear trend; Fig 3B), and at the global level the increase of cardiomyocyate size was reflected by an increase of LV mass-to-EDV across Weidemann categories (Fig 3C).

## Discussion

In this study, FRDA patients with preserved LVEF and without HF had a cardiac phenotype characterized by concentric LV hypertrophy in accordance with previous reports [15,22]. The expansion of ECV, combined with larger cell size (τ_ic_), reveals a tissue phenotype in FRDA characterized by interstitial fibrosis and cardiomyocyte hypertrophy, consistent with previous histological studies [4]. Novel methods of myocardial tissue phenotyping used in this study may help detect early signs of cardiomyocyte atrophy before a significant decline of LVEF and transition to heart failure. Taken together, these findings suggest that CMR characterization of the myocardial tissue phenotype provides novel insights into the cardiac disease severity in FRDA.

A cardiomyopathy was the underlying cause of death in 59% of patients with FRDA in a large retrospective study [23]. Despite the high burden of cardiac mortality, FRDA-CM has not received as much of a research focus as the neurological manifestations^6^. There are few published studies; most of them assessed small cohorts and solely employed echocardiographic data [24,25]. Recently, promising therapeutic agents for FRDA have emerged and some of them are moving towards human clinical trials [26,27]. Hence, there has been renewed interest in imaging methods to assess the FRDA-CM phenotype: first of all, because some of the potential drugs target specifically the heart [28]; secondly, because available neurological or neuroimaging biomarkers have proven disappointing to track longitudinal progression of the disease [29]. Taking these neurological scores as main outcome measure in randomized clinical trials proves difficult, since this would require excessively large sample sizes for such a rare disease. Therefore, we believe that the current study provides novel insights into the potential use of CMR-based biomarker of myocardial remodeling in FRDA. In particular, our results indicate CMR-derived parameters may help assess microstructural changes in the heart of patients with FRDA that appear to be associated with disease progression and neurological decline.

The intracellular water-lifetime, τ_ic_, has been validated by histological analyses as a non-invasive measure directly related to cardiomyocyte size [13]. It depends on the mean time for diffusion of water between the interstitial and intracellular spaces, which is closely related to cell size [13]. A higher than normal τ_ic_ indicates that cardiomyocyte hypertrophy is prevalent in FRDA-CM. τ_ic_ was approximately twice as long in FRDA-CM compared to controls, suggesting a doubling of cardiomyocyte size, and a 4 x larger cross-sectional area. The latter estimation for cardiomyocyte cross-sectional area is in excellent agreement with histological results from a previous study [30] of FRDA patients (cardiomyocyte cross-sectional area of 804 μm^2^ vs. 249 μm^2^ in controls). Patients with later onset, i.e. shorter disease duration when presenting for the CMR study, had higher τ_ic_ values. FRDA-CM generally first presents with cardiomyocyte hypertrophy, and in a later phase—to borrow a term from HCM—manifests itself by a regression of cardiomyocyte hypertrophy or possibly LV atrophy and LV dilation. This could potentially explain why patients diagnosed earlier, and with longer disease duration, have a lower τ_ic_ [15,28]. In mouse models of FRDA [28,31], myofibrills are atrophied and the initial cardiac hypertrophy has developed into a dilated cardiomyopathy at the onset of cardiac failure.

FRDA is associated with an increased risk of diabetes mellitus (DM). It has been previously established that DM is independently associated with worse functional status in FRDA [32], independently of GAA repeat length. In the present study we observed a worse neurological status in FRDA patients with DM, compared to non-diabetic subjects. The effects of FRDA-associated DM on the cardiac phenotype involved a lower cardiomyocyte size compared to non-diabetic subjects. The decreased expression of frataxin in FRDA patients has a profound impact on ATP production within mitochondria and is the primary cause of mitochondrial dysfunction in FRDA. As mitochondrial function plays an important role for the regulation of cell size [33], our finding of a smaller 1H intracellular lifetime provides novel mechanistic insights: a lower metabolic rate due to mitonchondrial dysfunction appears to have a limiting effect on cell-size. While LV hypertrophy has been considered a defining hallmark of Friedreich’s ataxia, the present finding suggests that disease progression, manifested by worse neurological status, and onset of DM, is associated with a regression of cardiomyocyte hypertrophy, possible as a result of worsening mitochondrial dysfunction.

ECV correlated with the classification of cardiac phenotype based on the criteria of Weidemann et al [15]. This classification ranks the expression of a cardiac phenotype based on intraventricular septal thickness and LV ejection fraction. Our study suggests that adverse myocardial tissue remodeling in the form of interstitial fibrosis and cardiomyocyte hypertrophy is associated with the Weidemann ranking, thereby providing some insight into mechanistic pathways in FRDA cardiomyopathy.

Our results are consistent with pathological analyses of the heart in FRDA [30,34]. Koeppen et al. assessed post-mortem data of 15 patients with FRDA and found a general pattern of concentric hypertrophy with reduced ventricular sizes, and thickened walls. On microscopy, fibrosis and collagen deposition were reported as conspicuous findings. There was also remarkable cardiomyocyte hypertrophy (mean area of 693μm^2^ x 250μm^2^ in controls). The authors hypothesized that such hypertrophy could be an adaptive response to fiber loss.

## Limitations

The current study has several limitations. Firstly, the observational cross-sectional design of this study did not allow a longitudinal analysis. While cross-sectional observations such as the association of CMR parameter with FARS3 scores and the age at the MRI exam are of interest and hypothesis-generating, only a longitudinal analysis can provide further insights on disease progression in FA. Secondly, is its limited sample size. Although we detected significant differences between FDRA patients and healthy controls, further investigations should be performed to further elucidate myocardial tissue remodeling during the progression to heart failure. Thirdly, we chose to use a Look-Locker approach rather than standard sequences (e.g., modified Look-Locker techniques) since this approach has been validated for quantification of τ_ic_. Moreover, standard T2-weighted images have not been acquired, despite proven useful for other cardiomyopathies. Fourthly, while healthy volunteers and FRDA subjects have been imaged using the same filed strength (3 Tesla) and the exact same imaging parameters, two different systems (FRDA subjects: Achieva, Philips Medical Systems, Best, The Netherlands and healthy volunteers: Siemens Trio, Siemens Medical Systems, Erlangen, Germany). Our reference values for healthy volunteers are in broad agreement with published reference values [16]. In addition, although the intracellular lifetime of water (τ_ic_) has been validated as a consistent marker of cardiomyocyte size [13] and subsequently applied in clinical studies [35,36], confirming its feasibility to independently estimate the cardiomyocyte size, results and conclusions obtained from this novel marker may be interpreted with caution. It is important to underline that the intracellular lifetime was not validated in human studies, as this would require access to endomyocardial biopsy samples obtained based on a clinical indication. A histological validation of intracellular lifetime in humans may change the interpretation of the results of the study, but the observed correlations of this novel tissue marker with global measures of LV hypertrophy indicate that cell hypertrophy is very likely to play a role in the observed changes of intra-cellular lifetime in FRDA patients. Finally, outcomes assessment was not possible because of the study design.

## Conclusions

LV mass index, ECV and τ_ic_ were notably elevated in FRDA patients even in the absence of LGE, consistent with a cardiac hypertrophy phenotype. CMR biomarkers of tissue remodeling from T1 mapping data provide novel insights into the myocardial tissue remodeling and cardiac disease severity in FRDA.
